# Droplet Digital PCR Assay for Detection and Quantification of ‘*Candidatus* Phytoplasma solani’ in Grapevine Samples

**DOI:** 10.3390/biology14091251

**Published:** 2025-09-11

**Authors:** Lucia Landi, Sergio Murolo, Gianfranco Romanazzi

**Affiliations:** Department of Agricultural, Food and Environmental Sciences, Marche Polytechnic University, Via Brecce Bianche, I60131 Ancona, Italy; l.landi@univpm.it (L.L.); s.murolo@univpm.it (S.M.)

**Keywords:** Bois noir, *Candidatus* Phytoplasma solani, droplet digital PCR, quantitative PCR, *Vitis vinifera* L.

## Abstract

‘*Candidatus* Phytoplasma solani’ is the causal agent of the Bois noir (BN), affecting grapevine worldwide. The complex epidemiology of BN, which involves multiple ‘*Ca*. P. solani’ host plants and insect vectors, as well as the occurrence of recovery (loss of symptoms on grapevine canopy), makes disease investigations and containment in vineyards difficult. To achieve early detection of ‘*Ca*. P. solani’, a droplet digital PCR (ddPCR)-based approach and quantitative (q)PCR assay were compared, testing specific primers based on the elongation factor Tu (*tuf*) gene using SYBR Green chemistry. The regression curve analysis of the ddPCR assay showed good linearity. Compared with the qPCR method, the sensitivity of ddPCR improved about 10-fold. The analysis of grapevine roots spiked with serial dilutions of ‘*Ca* P. solani’. PCR *tuf* fragments showed that qPCR was inhibited, while ddPCR was not affected. Testing 66 grapevine samples from 50 grapevine plants, the ddPCR provided superior diagnostic performance compared to qPCR in roots of symptomatic plants (75% detected by ddPCR, 41.6% by qPCR), roots of recovered plants (58.8% detected by ddPCR, 25% by qPCR), and asymptomatic leaf tissues from recovered plants (75% detected by ddPCR, 25% by qPCR). The ddPCR analysis allowed us to detect ‘*Ca*. P. solani’ on 40% of leaf samples from recovered plants and 20% of roots from asymptomatic plants. No differences among ddPCR and qPCR were found in detecting phytoplasma on symptomatic leaf samples. The ddPCR assay allowed the absolute quantification of ‘*Ca*. P. solani’ in complex matrices, such as roots, and when low titer of phytoplasma is present.

## Simple Summary

‘*Candidatus* Phytoplasma solani’ is the phytoplasma responsible for grapevine Bois noir (BN)s. BN is a complex disease that affects the plant’s phloem and is transmitted by insect vectors that feed on weeds (phytoplasma reservoirs) and occasionally move to healthy grapevines. Infected vines can clearly show symptoms but may undergo lost canopy symptoms (recovered plants), still harboring low levels of the pathogen, often in their roots. Therefore, a sensitive molecular technique is required for the early detection of the phytoplasma, with sensitivity at very low concentrations. In this study, we developed and compared two quantitative Polymerase Chain Reaction (qPCR) methods: droplet digital PCR (ddPCR) and standard qPCR. The ddPCR assay was found to be ten times more sensitive than standard qPCR and less affected by PCR inhibitors present in various plant matrices. When 66 grapevine samples were analyzed, ddPCR showed greater sensitivity than qPCR in detecting phytoplasma in the roots of symptomatic and recovered plants, as well as in asymptomatic leaf tissue of recovered plants. No significant difference in sensitivity was observed between the methods in symptomatic leaf samples. Overall, ddPCR proved useful for analyzing complex plant tissues with low pathogen concentrations.

## 1. Introduction

‘*Candidatus* Phytoplasma solani’ (taxonomic subgroup 16SrXII-A), order Acholeplasmatales, class Mollicutes [1], is the causal agent of Bois noir (BN), an important disease affecting grapevines (*Vitis vinifera*), which significantly reduces yield and causes economic losses in all major wine-growing areas [2,3,4]. BN has been reported in the Mediterranean region and the southern half of Europe [5,6,7]. It has occasionally been reported in other countries EPPO 2025 [8].

‘*Ca*. P. solani’ reveals a complex epidemiology [9,10,11,12] and is acquired by polyphagous vectors [5,13,14], which are able to feed and transmit infections on a wide spectrum of host plants [15,16,17] including grapevines that are dead-end hosts [5,18].

Grapevine BN symptoms include leaf yellowing or reddening (in white and red cultivars, respectively), downward leaf curling, flower abortion, berry shriveling, irregular ripening, and plant decline [2,3]. However, the phytosanitary control of this important disease in Europe is complex and involves numerous factors linked to the management of host plants, insect vectors, and the use of plant propagation material [19,20]. A key component of effective disease management is timely diagnosis, which enables early detection and containment. The optimal period for the diagnosis of ‘*Ca*. P. solani’ in grapevine in the Northern Hemisphere is generally from June to September, coinciding with the manifestation of BN symptoms on the leaves before harvesting. However, occasionally, phytoplasma has been reported in trunk, cordon, and shoots of grapevine affected by BN [21] or asymptomatic grapevine [22]. Furthermore, an attractive aspect of the epidemiology of BN is the process of ‘recovery’, which is the spontaneous disappearance of BN symptoms from previously symptomatic plants [23], but weak amplicons of phytoplasma were reported in the canopy [24] and roots [25]. In addition, the variable distribution and the low concentration of phytoplasmas on woody plants [2] makes detection with the most common molecular techniques difficult. Traditional molecular detection methods have focused on analyzing housekeeping or membrane protein genes. These approaches typically involve PCR followed by restriction fragment length polymorphism (RFLP) and amplicon sequencing [9,16,26,27,28]. In recent years, more advanced techniques such as quantitative real-time PCR (qPCR) [29,30,31] and high-resolution melting analysis combined with qPCR (HRM-qPCR) [25] have also been developed.

Droplet digital PCR (ddPCR) technology may offer increased sensitivity for the quantitative detection of ‘*Ca*. P. solani’. The ddPCR is based on the principle of partitioning the sample into several PCR sub-reactions using ‘water-in-oil’ droplets containing single, few, or no target sequences. The PCR partitions are read and counted as negative or positive based on their fluorescence amplitude, using Poisson’s statistics method [32]. Compartmentalization reduces the effect of amplification efficiency and PCR inhibitors, including those found in complex matrices, by mitigating template competition [33,34]. In ddPCR, a single sample is partitioned into thousands of droplets, with each droplet acting as a technical replicate. This process allows for the absolute quantification of target DNA, reducing the necessity for traditional technical replicates. Unlike qPCR, which relies on a standard curve for relative quantification, ddPCR provides a more precise, absolute count of molecules, which also helps to reduce pipetting errors [35,36]. Although quantitative real-time PCR (qPCR) is widely used for the detection of ‘*Ca*. P. solani’, its sensitivity can be limited, particularly in samples with low phytoplasma titer or uneven pathogen distribution. In this context, ddPCR is a promising alternative, providing higher sensitivity and thereby enhancing the reliability of ‘*Ca*. P. solani’ detection in low-titer samples. In recent years, ddPCR technology has been gradually applied in the detection of several pathogens [37,38]. The use of ddPCR based on TaqMan chemistry has also been proposed for phytoplasma [39]. Both qPCR and ddPCR were used to detect 16SrIV-D phytoplasma in different leaf tissues from common ornamental palms [40]. Compared to loop-mediated isothermal amplification (LAMP) technology, the sensitivity of ddPCR for detecting palm yellow leaf phytoplasma was improved by about 1000 times, detecting as few as 0.07 copies/µL [41]. In our work, we propose the ddPCR approach utilizing SYBR Green chemistry and dsDNA binding dye for the detection of ‘*Ca* P. solani. In ddPCR, SYBR Green chemistry offers several advantages over TaqMan probes, including lower cost, simpler assay design, and faster optimization. These features are particularly beneficial when targeting organisms with high sequence variability, such as phytoplasmas, which often inhabit a wide range of host species. The use of SYBR Green eliminates the need for specific probe binding sites, making it more adaptable to diverse genetic variants of ‘*Ca*. P. solani’. Although SYBR Green lacks the specificity and multiplexing capacity of probe-based systems, the ddPCR partitioning mechanism helps mitigate non-specific amplification, enabling sensitive and reliable detection when assays are carefully optimized [42].

Then the aim of the work was to set up a protocol based on ddPCR technology using SYBR Green chemistry for ‘*Ca*. P. solani’ detection and compare this technology with qPCR to detect ‘*Ca*. P. solani’ in leaves and roots from symptomatic, recovered, and asymptomatic grapevine.

## 2. Materials and Methods

### 2.1. Plant Sources

The protocols were validated on DNA extracted from a P7 calibrator sample obtained from P7 isolate phytoplasma inoculated on periwinkle plants, kindly provided by Dr. Xavier Foissac (INRA and University of Bordeaux, France). We analyzed 66 grapevine field samples for the presence of ‘*Ca*. P. solani’. The samples were collected from 50 different ‘Chardonnay’ plants in the Marche region. The samples included tissues from symptomatic, asymptomatic, and recovered plants. The analyzed samples were grouped as follow: (a) symptomatic leaves from symptomatic plants (21 samples); (b) asymptomatic leaves from symptomatic plants (4 samples); (c) asymptomatic leaves from recovered (5 samples) and asymptomatic plants (6 samples); (d) roots from symptomatic plants (12 samples); and (e) roots from recovered (12 samples) and asymptomatic plants (6 samples) (Table 1, Table 2, Table 3 and Table 4).

### 2.2. DNA Extraction

Total DNA extraction was performed as reported by Landi et al. [25]. Briefly, 2 g of pooled roots or leaf tissue was ground in liquid nitrogen. For each sample, about 200 mg of the powdered materials was collected and mixed with cetyl trimethyl ammonium bromide (CTAB) extraction buffer (3% CTAB, 100 mM Tris-HCl, pH 8.0, 20 mM EDTA, 1.4 M NaCl, 2% [*w*/*v*] soluble PVP-40) in a 2 mL tube then incubated at 68 °C for 30 min. The purification was performed on chloroform/isoamyl alcohol (24:1) and the precipitation on 0.6% isopropanol. After the 50 μL pure sterile water dissolved, the DNA purity and quantification were assessed by BioPhotometer plus (Eppendorf Inc., Westbury, NY, USA) with the absorption ratios at 260/280 in the range of 1.6–1.8, and at 260/230 in the range of 1.3–2.0.

### 2.3. Quantitative PCR Assay

The high-resolution melting (HRM) qPCR protocol reported by [25] was modified using suitable reagents for qPCR.

Briefly, for all qPCR reactions, the mix was prepared in a final volume of 10 µL containing 5 μL of 2× SsoAdvanced Universal SYBR Green Supermix (Bio-Rad Laboratories, Hercules, CA, USA), 4.8 µL of DNA, and 0.1 µL at a concentration of 25 µM of each primers, forward Tuf-U/f (5′-GATCCAGTGCGTGAAGTTGA-3′) and reverse Tuf-U/r (5′-ATTCCACGCAACAAAGCTCC-3′), [25] with the same quantity of primers set up for ddPCR (see below). For grapevine samples (leaves, roots), 10 ng total DNA/reaction was used as the template. Positive and negative amplification controls were included in each test. The amplification conditions were 95 °C for 3 min, followed by 40 cycles of 95 °C for 15 s and 60 °C for 50 s. The final step includes melting curve analyses (0.5 °C step increments; 10 s hold before each acquisition), which were conducted from 70 °C to 95 °C. Tests were performed on the thermal cycler CFX96 Touch Real-Time PCR Detection System (Bio-Rad, Hercules, CA, USA) using 96-well plates (Bio-Rad). The qPCR amplification efficiency was estimated for each matrix from the slopes of the standard curves generated by the 10-fold serial dilutions, using the equation E = 10 (−1/slope) − 1.

### 2.4. Droplet Digital PCR Assay

Droplet digital PCR was performed on the QX200 Droplet Digital PCR System (Bio-Rad) according to the manufacturer instructions [42,43]. To achieve a better separation of positive and negative droplet clusters without ‘droplet rain’, the DNA calibrator sample (P7) was tested at different amounts (10^−1^, 10^−2^, 10^−3^ ng) and two different primer concentrations (250 nM; 500 nM) in 20 µL of the reaction mix (2× QX200^TM^ ddPCR^TM^ EvaGreen Supermix) (Bio-Rad). In detail, the reaction mix (20 μL) and 70 μL of droplet-generating oil (Bio-Rad) [42,43] were added to a droplet-generating DG8 cartridge and loaded onto the Bio-Rad Automated Droplet Generator (Bio-Rad). The water-in-oil droplets (40 µL) were transferred to a 96-well PCR plate, heat-sealed at 180 °C with a pierceable foil using a PX1TM PCR Plate Sealer (Bio-Rad), and then placed in a C1000 Thermal Cycler (Bio-Rad) for end-point PCR. Amplification reactions were performed in the thermal cycler ICycler (Bio-Rad) with a ramp rate of 2 °C/s with the following protocol: 95 °C for 5 min followed by 40 cycles of denaturation at 95 °C for 30 s and annealing/extension at 58°C or 60 °C, respectively, for 1 min. The reaction was stopped at 4 °C for 5 min followed by 90 °C for 5 min. After ddPCR optimization, the 250 nM primers concentration and 60 °C annealing/extension were used for analyzing all samples tested. Then, 10 ng of total DNA templates was used to analyze the grapevine plant materials. Positive and negative amplification controls were included in each test. After amplification, the PCR plate was transferred to the droplet reader (QX200TMDroplet DigitalTM System (Bio-Rad)) set in the absolute quantification (ABS) modality. This incorporates the calculation of the basic parameters of the ddPCR (i.e., concentration, mean amplitudes of positive and negative droplets), the mean copies per partition and the total volume of the partitions measured, as defined by the digital MIQE guidelines [43,44]. Only the reactions with more than 12,000 validated droplets were considered in the analysis [42].

### 2.5. Assays Linearity, Limit of Detection (LOD), and Limit of Quantitation (LOQ)

The P7 calibrator sample was used to evaluate the linearity, the limit of detection (LOD), and the limit of quantitation (LOQ) by comparing the ddPCR and qPCR systems. For high-precision applications, the LOQ and LOD represent the lowest concentration at which all replicates are amplified with a coefficient of variation (CV) or of Cq or copy number values ≤ 25%, in accordance with MIQE guidelines. These metrics define the point at which an assay can reliably detect a target (LOD) and accurately quantify it (LOQ) [35,43]. The 10-fold serial dilutions (from 1.44 × 10^−2^ ng/reaction to 1.44 × 10^−9^ ng/reaction) of purified *tuf* gene PCR amplicon obtained by P7 calibrators, 242 bp (corresponding on average value ranging from 5.51 × 10^7^ to 5.51 × 10^0^ copies/reaction) (https://www.technologynetworks.com/tn/tools/copynumbercalculator (URL accessed on 11 February 2025)) [45], were analyzed. The possible interference of plant matrices was also investigated using the DNA of healthy grapevine root matrix (quantity 250 ng; quality by Nanodrop 8000 UV-VIS spectrophotometer: 260/280 1.8; 260/230 1.5) spiked with serial dilution of the qPCR amplicon of the P7 calibrators previously described. The LOD was also calculated according to 10-fold serial dilutions (from 1 to 1 × 10^−5^ ng/μL) of DNA extracted by the P7 calibrator sample. The qPCR linearity of performance was estimated for each matrix from the slopes of the standard curves generated by the 10-fold serial dilutions generated by CFX™ Maestro Software version 2.2 (Bio-Rad). For the ddPCR experiment, the linearity of the system’s performance was estimated by linear regression analysis performed via Excel. Finally, the appropriate quantity of DNA template was also verified with 5-fold serial dilution of DNA extracted from symptomatic leaves (samples MV18 and P2) and roots from symptomatic plants (S-y-5/6). For qPCR experiments, 8 technical replicates were performed while 4 technical replications were performed for ddPCR. The CV was calculated as the standard deviation of the data by the mean and then the result was multiplied by 100 (acceptable value ≤ 25%).

## 3. Results

### 3.1. Assessment of Linear Dynamic Range and Precision of ddPCR and qPCR Techniques

**Figure 1:**
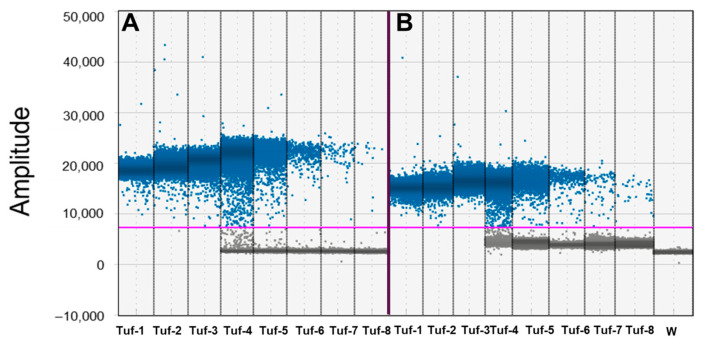
Droplet digital PCR (ddPCR) performance on serial dilutions of P7 *tuf* PCR fragment of ‘*Candidatus* Phytoplasma solani’, alone or with grapevine root spiked sample. Performance of droplet digital PCR (ddPCR) related to 10-fold serial dilutions of purified *tuf* qPCR amplicon from P7 calibrator sample obtained from P7 ‘*Ca*. P. solani’ isolate inoculated on periwinkle plants, alone (Tuf-1_Tuf-8; from 1.44 × 10^−2^ to 1.44 × 10^−9^ ng/reaction) (**A**) or combined with 250 ng DNA from grapevine roots from healthy plants (**B**). Ordinate scales indicate fluorescent amplitude. Pink line is threshold, above which are positive droplets (blue) containing at least one copy of target DNA and below which are negative droplets (gray) without any target DNA. Samples are divided by vertical dotted yellow line. Tuf-1, Tuf-2, and Tuf-3 are saturated samples (see manuscript for details). W = water.

The optimal primer concentrations and annealing/extension temperature of 250 nM and 60 °C, respectively, were set up. These reaction conditions proved to be suitable for detecting ‘*Ca*. P. solani’ in all matrices analyzed, with a significant difference in the fluorescence signals between positive and negative droplets, generating a high number of amplification products (number of positive droplets). The qPCR and ddPCR sensitivity comparison using a template qPCR *tuf* fragment generated by the P7 calibrator sample showed that ddPCR was more sensitive than qPCR by 10-fold. The LOD for qPCR was observed at a Cq value of 33.9 ± 0.86, corresponding to the Tuf-7 sample. This translates to 1.44 × 10^−8^ ng of DNA per 20 µL reaction, which is equivalent to 5.51 × 10^1^ copies per reaction. In contrast, the LOD for ddPCR was the Tuf-8 sample, containing 1.44 × 10^−9^ ng of DNA per 20 µL reaction (approximately 5.51 × 10^0^ copies per reaction). The expected copy number for the Tuf-8 sample closely matched the ddPCR results, which showed 5.7 ± 0.6 copies per 20 µL reaction. This corresponds to the ddPCR limit of quantification (LOQ) for ‘*Ca*. P. solani’ using this approach (Table 1; Figure 1A).

DdPCR yielded a reduced linearity range compared to qPCR. For example, in ddPCR, the reactions were positively saturated in samples Tuf-1, Tuf-2, and Tuf-3 (from 1.44 × 10^2^ to 1.44 × 10^4^ ng), whereas the same samples were still detectable by qPCR (Table 1 and Figure 1A). qPCR analysis of the spiked samples indicated the presence of inhibitors, with the standard curve showing an unrealistic PCR efficiency over 100% (Efficiency 142.5%; slope –2.695; R^2^ 0.925) (Table 1).

A significant linear calibration curve was generated by serial dilution of the P7 purified *tuf* qPCR amplicon (Efficiency 97.9%; slope –3.372; R^2^ 0.999) (Table 1). Linear regression analysis showed that ddPCR was not affected by the inhibitory action of PCR interferers associated with the high quantity of DNA from the spiked root samples. The linear regression was y = 4 × 10^9^x + 414.33 for the P7 purified *tuf* qPCR amplicon and y = 4 × 10^9^x + 347.09 for the spiked samples, which provides evidence of no interference on the LOQ (Table 1). However, the fluorescence signals for positive droplets in the spiked samples show an amplitude typically inferior to 20,000, with a minor difference between them and the negative droplets (Figure 1B).

**Table 1:** Assessment of linear dynamic range and precision of ddPCR and qPCR techniques. Concentration represents 10-fold serial dilutions of purified *tuf* qPCR amplicon from P7 alone or combined with 250 ng DNA from grapevine leaf from healthy plants. Results were related to eight replicates for qPCR and four for ddPCR. Mean ± standard deviation and CV are shown.

Purified *tuf* qPCR Amplicon from Periwinkle Inoculated with P7 Isolate (P7)									
**ID** **Sample**	**DNA (ng**)	qPCR (Cq) Mean ± SD		qPCR CV (%)		ddPCR (Copies Per 20 μL/Reaction) Mean ± SD		ddPCR CV(%)	
		P7	P7+ 250 mg of DNA from Roots	P7	P7+ 250 mg of DNA from Roots	P7	P7+ 250 mg of DNA from Roots	P7	P7+ 250 mg of DNA from Roots
Tuf-1	1.44 × 10^−2^	13.6 ± 0.37	15.4 ± 0.24	3.58	1.94	saturated	saturated	nd	nd
Tuf-2	1.44 × 10^−3^	16.7 ± 0.30	18.9 ± 0.32	2.2	1.82	saturated	saturated	nd	nd
Tuf-3	1.44 × 10^−4^	19.9 ± 0.43	21.6 ± 0.20	2.4	1.02	saturated	saturated	nd	nd
Tuf-4	1.44 × 10^−5^	23.3 ± 0.2	25.6 ± 0.16	0.94	0.68	52,340 ± 1126	54,067 ± 2338	2.15	4.32
Tuf-5	1.44 × 10^−6^	26.5 ± 0.49	28.4 ± 0.24	1.99	0.86	6871 ± 126.7	6755 ± 141.1	1.84	2.06
Tuf-6	1.44 × 10^−7^	31.3 ± 0.55	29.5 * ± 0.25	1.97	0.84	687 ± 37.5	584 ± 28.9	6.058	4.84
Tuf-7	1.44 × 10^−8^	33.9 ± 0.86	30.5 * ± 036	2.78	1.20	65± 8.0	64 ± 10.4	11.51	16.13
Tuf-8	1.44 × 10^−9^	−	−	−	−	5.7 ± 0.6	7.1 ± 0.8	11.21	11.33
Statistics of qPCR: Standard Curve Performance						Statistics of ddPCR: Linear Regression			
		Slope		−3.372	−2.605	y = 4 × 10^9^x ± 414.33		y = 4 × 10^9^x ± 347.09	
		Value of fit (R^2^)		0.999	0.925	Value of fit (R^2^)		0.999	R^2^ 0.9993
		Efficiency		97.9%	142.5%				

Note: Cq = threshold cycle values obtained by qPCR. CV (%) = standard deviation/average, used to analyze precision of detection work; CV (%) ≤ 25%, indicated this system has good repeatability [35,43]. * = sample not amplified; nd = not determined (see manuscript for details).

**Figure 2:**
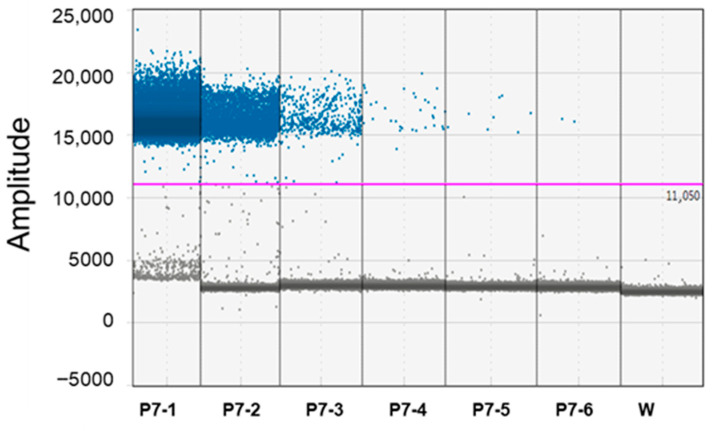
Performance of droplet digital PCR (ddPCR) related to 10-fold serial dilutions of P7 calibrator sample obtained from P7 ‘*Candidatus* Phytoplasma solani’ isolate inoculated on periwinkle plants. P7 calibrators as tested at different concentrations (P7-1_P7-6, from 1 × 10^−0^ to 1 × 10^−5^ ng/reaction). Ordinate scales indicate fluorescent amplitude. Pink line is threshold, above which are positive droplets (blue) containing at least one copy of target DNA and below which are negative droplets (gray) without any target DNA. W = water (see manuscript for details).

The 10-fold sensitivity difference between qPCR vs. ddPCR was also demonstrated with serial dilution of the P7 calibrator sample (Table 2; Figure 2).

**Table 2:** Assessment of linear dynamic range and precision of ddPCR and qPCR techniques. Concentration represents 10-fold serial dilutions of Periwinkle infected by ‘*Candidatus* Phytoplasma solani’ for P7 isolate. Results were related to eight replicates for qPCR and four for ddPCR. Mean ± standard deviation and CV are shown.

Periwinkle Infected by ‘*Candidatus* Phytoplasma solani’ for P7 Isolate					
ID Sample	Total DNA (ng)	Cq (qPCR) Mean ± SD	CV (%) (qPCR)	Copies Per 20 μL Well (ddPCR) Mean ± SD	CV (%) (ddPCR)
P7-1	1	22.0 ± 0.1	0.44	80,773 ± 5216	6.46
P7-2	10^−1^	24.8 ± 0.15	0.60	9195.5 ± 256	2.82
P7-3	10^−2^	28.3 ± 0.32	1.09	948.3 ± 51	5.40
P7-4	10^−3^	31.7 ± 0.20	0.64	104.6 ± 9.7	8.66
P7-5	10^−4^	34.1 * ± 0.32	0.58	19. ± 1.9	10.52
P7-6	10^−5^	−	−	3.4 ± 0.31	9.36
	Statistics of qPCR: standard curve performance Slope: −3.226 Efficiency: 104.2 Value of fit (R^2^): 0.987			Statistics of ddPCR: linear regression y = 72,554x + 233.97 Value of fit R^2^ = 0.9998	

Note: Cq = threshold cycle values obtained by qPCR. CV (%) = standard deviation/average, used to analyze the precision of detection work; CV (%) < 25%, indicated this system has good repeatability [35,43]. * = positive five replicated of eight analyzed.

**Figure 3:**
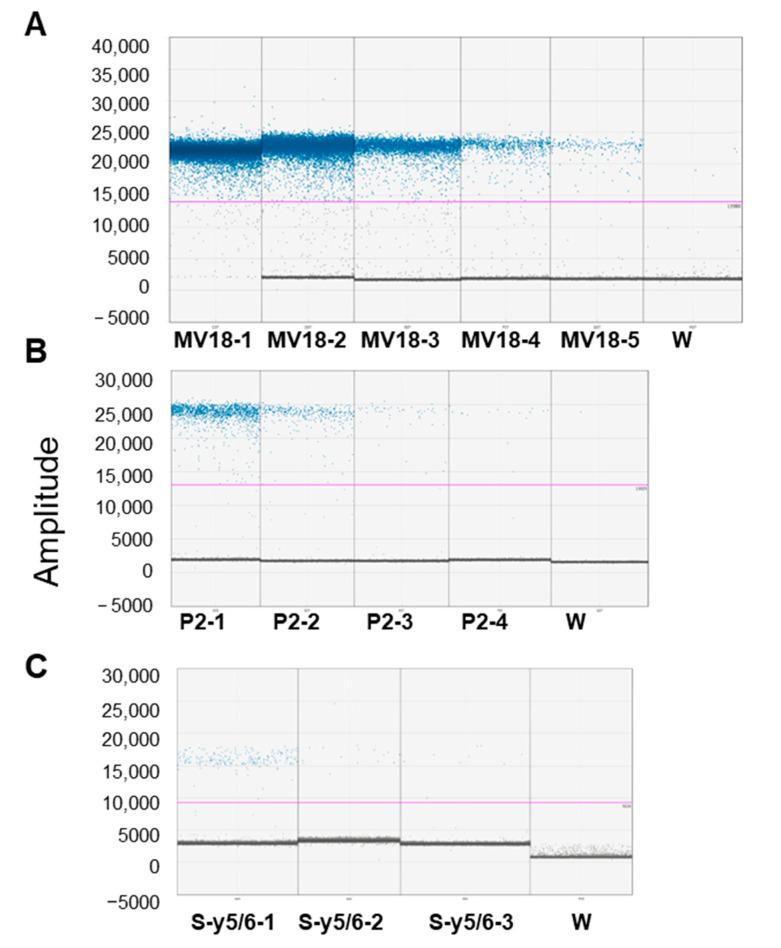
Droplet digital PCR (ddPCR) performance to detect ‘*Candidatus* Phytoplasma solani’ on leaves and roots from symptomatic plants. DdPCR detection related to 5-fold serial dilutions of ‘*Ca*. P. solani’ on symptomatic leaf tissue samples, (**A**) MV18 samples, (MV18-1_MV18-5, from 200 to 0.4 ng DNA ng/rection), (**B**) P2 (P2-1_P2-4, from 40 to 1.6 DNA ng/rection), (**C**) and roots from symptomatic plants (Sy-5/6) (from 50 to 2 DNA ng/rection). Ordinate scales indicate fluorescent amplitude. Pink line is threshold, above which are positive droplets (blue) containing at least one copy of target DNA and below which are negative droplets (gray) without any target DNA. W = water (see manuscript for details).

The analysis of serial dilution of grapevine samples from symptomatic plants suggests that within a range of 200–0.08 ng of total DNA used as the template, the ability to detect the ‘*Ca*. P. solani’ *tuf* gene by ddPCR is independent of the DNA concentration but must be calibrated in relation to the phytoplasma concentration (Table 3 and Figure 3A–C). Indeed, saturation phenomena at very high phytoplasma concentrations were observed (Figure 3A). The CVs that were determined for repeatability among the ddPCR replicates and for reproducibility among the independent experiments were much lower than the acceptance criterion of ≤25% [35,43].

**Table 3:** Comparison of ddPCR and qPCR techniques on detecting *Ca*. P. solani’ on symptomatic leaf tissue samples (MV18, P2) and roots from symptomatic plants (S-y5/6). Results were related to four replicates for qPCR and two for ddPCR. Mean ± standard deviation and CV are shown.

Symptomatic Leaf Tissue Samples (MV18, P2) and Roots from Symptomatic Plants (S-y5/6)					
ID Sample	Total DNA (ng)	Cq (qPCR) Mean ± SD	CV (%) (qPCR)	Copies Per 20-μL Well (ddPCR) Mean ± SD	CV (%) (ddPCR)
MV18-1	50	22.2 ± 0.3	1.33	saturated	−
MV18-2	10	23.8 ± 0.34	1.48	37,572 ± 643.2	1.71
MV18-3	2	24.2 ± 0.41	0.94	5540 ± 370.4	6.68
MV18-4	0.4	27.0 ± 0.28	0.65	1230 ± 101.5	8.25
MV18-5	0.08	30.2 ± 0.34	0.81	279 ± 31	11.09
P2-1	200	27.9 ± 0.21	0.86	1487 ± 170.6	11.47
P2-2	40	29.3 ± 0.31	1.19	287 ± 25.98	9.070
P2-3	8	31.1 ± 0.75	2.67	73 ± 5.68	7.82
P2-4	1.6	32.8 ± 0.51	1.73	24 ± 3.62	15.02
S-y5/6-1	50	30.1 ± 0.45	1.51	190 ± 20.04	10.52
S-y5/6-2	5	31.9 ± 0.51	1.62	34 ± 3.6	10.6
S-y5/6-3	2	−		8 ± 1.5	19.9

### 3.2. ‘Ca. P. solani’ Detection on Grapevine Samples: ddPCR vs. qPCR

The analysis of symptomatic, recovered, and asymptomatic grapevine samples highlights a greater ability of ddPCR vs. qPCR to detect ‘*Ca*. P. solani’ in asymptomatic tissue, while no difference was observed for symptomatic tissue. The detection by qPCR and ddPCR revealed ‘*Ca*. P. solani’ in all twenty-one DNA samples extracted from symptomatic leaf tissue of symptomatic plants. The absolute quantification of ‘*Ca*. P. solani’ performed by ddPCR ranged from 176.4 ± 67 copies/reaction in sample SC10 to 37,572 ± 453 copies/reaction for sample MV18 in symptomatic leaf tissue (Table 4).

The analysis of asymptomatic leaf tissue from symptomatic plants showed ‘*Ca*. P. solani’ in one out of four samples with qPCR (25%) and in three out of four analyzed (75%) by ddPCR. The titer of ‘*Ca*. P. solani’ ranged from 9 ± 1.2 copies/reaction in sample P2 to 188 ± 16 copies/reaction in sample P4. The roots of twelve symptomatic plants were analyzed, with five being positive by qPCR (41.6%) vs. nine by ddPCR (75%). ‘*Ca*. P. solani’ ranged from 35 ± 4.3 copies/reaction in sample S-y5/6 to 898 ± 89 copies/reaction in sample S-y5/5. Concerning the asymptomatic leaf tissue from recovered plants, we detected the phytoplasma in two out of five plants analyzed (40%) only by ddPCR assay (ranging from 9.8 ± 2.2 copies/reaction in sample R-y5/3 to 13 ± 3.3 copies/reaction in sampleR-y5/2). Regarding detection in DNA root samples of twelve recovered plants, qPCR revealed the presence of phytoplasma in three plants (25%) while ddPCR in seven plants (58.5%). ‘*Ca*. P. solani’ ranged from 16 ± 3 copies/reaction in sample R-y11/1 to 97 ± 12 copies/reaction in sample R-y2/7. Finally, the investigation also involved the roots of six asymptomatic plants. ddPCR analysis detected traces of phytoplasma in two out of the six plants (Table 4).

**Table 4:** Comparison of ddPCR and qPCR techniques on detecting ‘*Ca*. P. solani’ in symptomatic, recovered, and asymptomatic plants (S/R/A) *. Symptomatic leaf tissue (S *-leaf); roots from symptomatic plants (S *-root); asymptomatic leaf tissue from asymptomatic, symptomatic, and recovered plants (A *-leaf); and roots from recovered and asymptomatic plants (A *-root). Results were related to three replicates for qPCR and two for ddPCR. Mean ± standard deviation is shown.

			qPCR (Cq) Mean ±SD				ddPCR (Copies Per 20-μL Well) Mean ±SD			
N°	Plant Code	S/R/A *	S *-Leaf	S *-Root	A *-Leaf	A *-Root	S *-Leaf	S *-Root	A *-Leaf	A *-Root
1	P1	S	26.8 ± 0.52	ne	−	na	3836.8 ± 232	ne	0	na
2	P2	S	30.4 ± 0.48	ne	−	na	1492 ± 211	ne	9 ± 1.2	na
3	P3	S	28.4 ± 0.32	ne	−	na	622.4 ± 65	ne	17.8 ± 3	na
4	P4	S	27.7 ± 0.27	ne	30.2 ± 0.21	na	1296 ± 253	ne	188 ± 16	na
5	MG14	S	25.9 ± 0.12	ne	ne	na	11,276 ± 434	ne	ne	na
6	MG15	S	26.6 ± 0.65	ne	ne	na	4532 ± 121	ne	ne	na
7	MG16	S	33.1 ± 0.53	ne	ne	na	36.2 ± 2.5	ne	ne	na
8	MV16	S	30.1 ± 0.58	ne	ne	na	690 ± 32	ne	ne	na
9	MV18	S	23.8 ± 0.42	ne	ne	na	37,572 ± 453	ne	ne	na
10	MV4	S	30.8 ± 0.61	ne	ne	na	287 ± 23	ne	ne	na
11	P99	S	30.1 ± 0.38	ne	ne	na	324 ± 44	ne	ne	na
12	P105	S	30.8 ± 0.44	ne	ne	na	218 ± 12	ne	ne	na
13	NT4	S	26.6 ± 0.64	ne	ne	na	4566 ± 321	ne	ne	na
14	NT5	S	27.2 ± 0.32	ne	ne	na	2340 ± 234	ne	ne	na
15	SC10	S	31.5 ± 0.58	ne	ne	na	176.4 ± 67	ne	ne	na
16	S-y5/2	S	ne	29.9 ± 0.54	ne	na	ne	898 ± 89	ne	na
17	S-y2/11	S	ne	−	ne	na	ne	98 ± 7.2	ne	na
18	S-y3/2	S	ne	−	ne	na	ne	−	ne	na
19	S-y4/1	S	ne	−	ne	na	ne	−	ne	na
20	S-y4/3	S	ne	−	ne	na	ne	−	ne	na
21	S-y4/5	S	ne	29.8 ± 0.81	ne	na	ne	452 ± 54	ne	na
22	S-y4/9	S	29.8 ± 0.51	−	ne	na	818 ± 56	48 ± 5.3	ne	na
23	S-y5/4	S	29.7 ± 0.51	29.1 ± 0.53	ne	na	1452 ± 121	846.8 ± 89	ne	na
24	S-y5/5	S	30.2 ± 0.51	30.2 ± 0.82	ne	na	2899 ± 99	368.4 ± 23	ne	na
25	S-y5/6	S	30.1 ± 0.51	−	ne	na	786 ± 43	35 ± 4.3	ne	na
26	S-y5/7	S	27.2 ± 0.51	−	ne	na	2404 ± 188	192.8 ± 22	ne	na
27	S-y5/12	S	29.8 ± 0.51	29.1 ± 0.55	ne	na	1320 ± 201	543 ± 88	ne	na
28	R-y5/1	R	na	na	−	ne	na	na	13 ± 3.3	ne
29	R-y5/2	R	na	na	−	ne	na	na	9.8 ± 2.2	ne
30	R-y5/3	R	na	na	−	ne	na	na	0	ne
31	R-y5/6	R	na	na	−	ne	na	na	0	ne
32	R-y5/8	R	na	na	−	ne	na	na	0	ne
33	R-y1/9	R	na	na	ne	31.4 ± 0.43	na	na	ne	54 ± 7
34	R-y1/11	R	na	na	ne	−	na	na	ne	16 ± 3
35	R-y2/1	R	na	na	ne	31.2 ± 0.29	na	na	ne	88 ± 21
36	R-y2/2	R	na	na	ne	−	na	na	ne	−
37	R-y2/4	R	na	na	ne	30.9 ± 0.48	na	na	ne	97 ± 12
38	R-y2/7	R	na	na	ne	−	na	na	ne	58 ± 3.5
39	R-y2/12	R	na	na	ne	−	na	na	ne	30 ± 4.1
40	R-y3/6	R	na	na	ne	−	na	na	ne	−
41	R-y4/4	R	na	na	ne	−	na	na	ne	34 ± 6.1
42	R-y4/5	R	na	na	ne	−	na	na	ne	−
43	R-y4/6	R	na	na	ne	−	na	na	ne	−
44	R-y5/4	R	na	na	ne	−	na	na	ne	−
45	AS2	A	na	na	−	−	na	na	−	13 ± 2.3
46	AS10	A	na	na	−	−	na	na	−	24 ± 3.3
47	AS9	A	na	na	−	−	na	na	−	−
48	AS-DBL	A	na	na	−	−	na	na	−	−
49	AS-K3	A	na	na	−	−	na	na	−	−
50	AS-NT5	A	na	na	−	−	na	na	−	−
Total positive samples			21	5	1	3	21	9	5	9

ne = not evaluated; na = not assessable; − = negative.

## 4. Discussion

In this study, we used ddPCR assay to detect and quantify ‘*Ca*. P. solani’ and verified the performance, efficiency, and sensitivity in the detection of pathogens vs. qPCR when analyzing different matrices. ddPCR was 10-fold more sensitive than qPCR. This result is consistent with previous studies, which found ddPCR to be 10- to 100-fold more sensitive than qPCR for detecting various pathogens. Specifically, ddPCR demonstrated superior sensitivity for Flavescence dorée (FD) phytoplasma [46], *Ilyonectria liriodendri* in grapevine [47], *Clavibacter michiganensis* subsp. *michiganensis* in tomato [48], *Stagonosporopsis cucurbitacearum* in seeds of *Cucurbita maxima* [49], and *Verticillium* spp. in soil [50]. In contrast, one study reported that while ddPCR significantly improved analytical sensitivity for *Ralstonia solanacearum* on potato, its sensitivity was similar to qPCR for detecting *Erwinia amylovora* on Rosaceae plants [51]. ddPCR also showed higher analytical sensitivity than qPCR for *Xylella fastidiosa* on *Olea europea*, *Citrus sinensis*, and *Nerium oleander* matrices [52], and a slightly improved diagnostic sensitivity on *Quercus ilex* and *Polygala myrtifolia* [53]. Finally, ddPCR was less sensitive than qPCR in detecting *Leishmaniasis parasite* [54]. PCR-inhibitory compounds in the matrix of the field samples could be the primary cause of the reduced detection ability of qPCR [55].

Previously we explored the effect of different sample matrices on the detection of ‘*Ca*. P. solani’ using an HRM-qPCR system. Our samples, consisting of root and leaf tissue spiked with the *tuf* qPCR amplicon, suggested that high concentrations of matrices, particularly roots, may inhibit or otherwise affect phytoplasma detection [25].In this study, we tested high concentrations of spiked grapevine root samples. We chose this matrix because it contains humic acid substances, which are known to influence DNA polymerases used in PCR analysis [56]. There was no correlation between the Cq values of the spiked samples and those obtained from the purified *tuf* qPCR amplicon of isolate P7. Furthermore, we did not observe any target amplifications at the lowest dilution.

The non-linear correlation can be dependent from inhibitor titer and/or the elevated quantity of the DNA matrix [56,57], suggesting in this condition a lower reliability of the qPCR system for absolute quantification [35]. The same samples, analyzed by ddPCR, showed to not be affected in ‘*Ca*. P. solani’ detection, indicating that the performance of ddPCR is much less influenced by the matrix. Unlike qPCR, ddPCR allows for the absolute quantification of the target DNA by partitioning PCR reagents and applying the Poisson algorithm [32]. This approach may reduce the bias inherent in qPCR assays, where sample concentrations are calculated using an external calibration curve [35]. qPCR assay is an indirect method that determines the original concentration of the target by measuring fluorescent signals, which are directly proportional to the amount of PCR product. Factors that negatively affect PCR efficiency can make the extrapolation from fluorescent signals in qPCR inaccurate. For example, when only a single or a few target molecules are present, the qPCR efficiency may decrease due to a reduced probability of primers and polymerase binding to the target sequence. This phenomenon is known as a stochastic effect [58]. qPCR offers high sensitivity, speed, and cost-effectiveness for applications like pathogen detection [59,60] and gene expression analysis [61,62,63]. However, our data show that ddPCR provides greater sensitivity, accuracy, and precision for quantifying ‘*Ca*. P. solani, particularly at low pathogen concentrations. Conversely, high DNA target concentrations saturated the ddPCR system, yielding a narrower linearity range compared to qPCR. Therefore, it is necessary to confirm that the amount of the target DNA is within the measurement range before performing the experiment. Our tests on grapevine samples highlight that when examining the DNA from symptomatic leaves (or petioles), no difference between qPCR and ddPCR was recorded.

Our work is the first to demonstrate that ddPCR was more effective than qPCR for detecting ‘*Ca*. P. solani’ in asymptomatic grapevine tissues, even in roots or asymptomatic leaf portions of symptomatic plants. This aspect is very important because ddPCR is useful for detecting and quantifying phytoplasma in asymptomatic tissue more easily and reliably without resorting nested qPCR cycles [25]. The application of a ddPCR approach for the detection and quantification of ‘*Ca*. P. solani’ presents new opportunities. It enables the monitoring of the phytoplasma’s presence and titer before symptoms manifest or during the latent period, especially in propagative material in the nursery phase. As there is currently no direct treatment for phytoplasma diseases, the ability to detect the pathogen with greater sensitivity is a powerful tool that allows for more proactive and effective management strategies, thereby mitigating the economic impact of these diseases [64]. It is therefore clear that the ddPCR tool applied to ‘*Ca*. P. solani’ will allow for clarifying several aspects of BN epidemiology and improving the control of BN with an easier and exact tool. The titer and distribution of ‘*Candidatus* Phytoplasma pruni’ in cherries plants were analyzed by qPCR, revealing that the infection starting from the roots then colonizes the canopy. Severe symptom onset was associated with three to four orders of phytoplasma magnitudes more than mild symptoms [65]. These data agreed with our results that detected in roots from asymptomatic or recovered plants a high quantity of phytoplasma compared to canopy and the highest titer in the symptomatic leaves. The slow rate of colonization and symptom expression can also explain the different susceptibilities of cultivars to BN [66]. The association between phytoplasma titer and symptom severity is host-specific. In some cases, phytoplasmas are found near, but rarely within, symptomatic tissue, as seen with palm phytoplasma dieback [67]. In others, such as Flavescence dorée, the phytoplasma is present before or at the onset of symptoms [68]. The disease known as bois noir (BN) is of increasing significance worldwide. Having a tool capable of detecting and quantifying the pathogen with a sensitive assay would be a significant help.

## 5. Conclusions

This study aimed to develop and validate sensitive ddPCR assay to detect and quantify ‘*Ca*. P. solani’. Our results demonstrate that the ddPCR technology is more sensitive compared to qPCR and robust for detecting ‘*Ca*. P. solani’ in grapevine field samples with low titer pathogens and residual matrix inhibitors. Furthermore, it can provide an accurate and reproducible alternative to qPCR for diagnostic applications. ddPCR is an advanced methodological tool, which can facilitate measurement standardization useful for inter-laboratory comparisons.

## Data Availability

The corresponding author will provide the necessary data supporting the findings of this study upon a reasonable request. The authors are accountable for ensuring the continued availability of the data.
